# Folliculogenesis Is Not Fully Inhibited during GnRH Analogues Treatment in Mice Challenging Their Efficiency to Preserve the Ovarian Reserve during Chemotherapy in This Model

**DOI:** 10.1371/journal.pone.0137164

**Published:** 2015-09-01

**Authors:** Florence Horicks, Géraldine Van Den Steen, Sarah Houben, Yvon Englert, Isabelle Demeestere

**Affiliations:** 1 Research Laboratory on Human Reproduction, Université Libre de Bruxelles (ULB), Brussels, Belgium; 2 Gynecology and Obstetrics Department, Erasme Hospital, Brussels, Belgium; Qingdao Agricultural University, CHINA

## Abstract

As many chemotherapy regimens induce follicular depletion, fertility preservation became a major concern in young cancer patients. By maintaining follicles at the resting stage, gonadotropin-releasing hormone analogues (GnRHa) were proposed as an ovarian-protective option during chemotherapy. However, their efficacy and mechanisms of action remain to be elucidated. Mice were dosed with cyclophosphamide (Cy, 100–500mg/kg i.p) to quantify follicular depletion and evaluate apoptosis at different times. We observed a dose-dependent depletion of the follicular reserve within 24 hours after Cy injection with a mean follicular loss of 45% at the dose of 200mg/kg. Apoptosis occurs in the granulosa cells of growing follicles within 12 hours after Cy treatment, while no apoptosis was detected in resting follicles suggesting that chemotherapy acutely affects both resting and growing follicles through different mechanisms. We further tested the ability of both GnRH agonist and antagonist to inhibit oestrus cycles, follicular growth and FSH secretion in mice and to protect ovarian reserve against chemotherapy. Although GnRHa were efficient to disrupt oestrus cycles, they failed to inhibit follicular development, irrespective of the doses and injection sites (sc or im). Around 20% of healthy growing follicles were still observed during GnRHa treatment and serum FSH levels were not reduced either by antagonist or agonist. GnRHa had no effect on Cy-induced follicular damages. Thus, we showed that GnRHa were not as efficient at inhibiting the pituitary-gonadal axis in mice as in human. Furthermore, the acute depletion of primordial follicles observed after chemotherapy does not support the hypothesis that the ovary may be protected by gonadotropin suppression.

## Introduction

Long-term survival of young patients treated for cancer has significantly increased, due to the growing effectiveness of treatments. However, chemotherapy and radiotherapy may also damage healthy cells, inducing long-term side effects such as primary ovarian insufficiency [1]. Quality of life issues, including fertility, are therefore moving to the forefront of oncologists concerns. As result of delaying childbearing, many young cancer patients are childless at the time of diagnosis. For these patients, the prospect of future infertility is an additional source of emotional distress [2]. The development of fertility preservation procedures has a major impact on their future quality of life, providing new hope about their ability to conceive after cancer remission [3, 4].

Among the most gonadotoxic agents, alkylating agents such as cyclophosphamide (Cy) is commonly used as cytotoxic or immunosuppressive therapy for the treatment of haematological diseases, breast cancer, or immunological benign diseases. Basically, Cy induces cross-links into the DNA strands, interfering with cell division and leading to apoptosis. However, whether the reduction of the ovarian reserve, including mainly the primordial follicles, results from a direct or indirect effect of chemotherapeutic agents remains unclear. A dramatic reduction of the primordial follicles was observed in human ovarian tissue xenografted in mice 48 hours after Cy administration, suggesting a direct effect of the drug on the resting pool [5]. On the other hand, the loss of growing follicles, more sensitive to chemotherapy, leads to a rapid decline in anti-Müllerian hormone (AMH) level, which induces a massive recruitment of the primordial follicles calling the ‘burn out effect’ [6, 7]. Recent data suggest that chemotherapy itself may induce massive recruitment of resting follicles into the growing pool of follicles by activation of the PI3K/PTEN/Akt pathway [8]. Altogether, the mechanisms of chemotherapy toxicity on reproductive cells remains under investigation and their better understanding is essential in order to develop new strategies for fertility preservation.

Pharmacological ovarian protection that aims to reduce the gonadotoxic effects of the chemotherapy appears to be a particularly attractive approach to preserve fertility [9]. Among these options, gonadotropin-releasing hormone analogues (GnRHa), including agonist and antagonist, may be suitable for protecting the ovaries from chemotherapy-induced damage by suppressing ovarian function. GnRHa are used since decades in assisted reproductive techniques for their inhibitory effect on the endogenous LH surge during controlled ovarian stimulation [10]. Their ability to inhibit the pituitary-gonadal axis is also useful in endometriosis, uterine fibroids and early onset puberty [11]. The ovarian protective effect of GnRHa against alkylating agent-induced damages has been reported in mice [12, 13], rat [14–18], and monkey [19]. However, others failed to demonstrate any ovarian protection by the use of GnRHa during chemotherapy [20, 21] and even showed toxic effects [22]. In humans, randomized prospective trials have also reported conflicting data [4, 23–25]. Furthermore, the potential mechanisms that mediate the protective effects remain unclear. The main hypothesis is based on the down-regulation action of GnRHa on the hypothalamic–pituitary–ovarian axis, maintaining follicles at resting stages that are thought to be more resistant to chemotherapy [26, 27]. By inducing various degrees of ovarian inhibition, GnRH agonist and antagonist may have a different protective effect [22, 28, 29]. While the inhibitory effect of GnRHa on folliculogenesis is not clearly demonstrated in mice, this model is commonly used to investigate the ovarian protective effect of GnRHa.

Hence this study was designed to evaluate the acute effect of Cy on the follicular pool and compare the efficacy of both GnRH agonist and antagonist in the prevention of chemotherapy-induced ovarian damage. In order to validate the model, we investigate the inhibitory effect of a GnRH agonist and antagonist at different doses on folliculogenesis, oestrus cycles, and follicle-stimulating hormone (FSH) levels in mice.

## Materials and Methods

All experiments were approved by the Animal Ethics Committee of the Medicine Faculty at the Université Libre de Bruxelles. Animals were maintained under standard conditions of light (12 hours light/dark cycle) and temperature in conventional facility and kept with maximum 10 animals per cage. They had access to food and water *ad libitum* and all efforts were made to minimize suffering of the animals following institutional guidelines. A delay of 5 days of accommodation to experimental conditions was mandatory before starting experiments. All animals were randomly allocated to each experimental group and were housed in groups according to treatments.

### Mice treatments

Hybrid female F1 (C57BL/6j x CBA/Ca) mice aged 5–8 weeks received a single intraperitoneal (ip) injection of Cy (Sigma-Aldrich, Belgium) at doses of 100, 200, and 500 mg/kg, or saline solution (control) (6 mice per condition). At least three mice treated with 200mg/kg of Cy were sacrificed at different time points between 1 hours and 21 days later in order to evaluate the dose and time-related damages on the follicular pool. Based on the results, the dose of 200mg/kg of Cy was chosen to analyse the ovarian protective effect of GnRHa.

Then, hybrid female F1 (C57BL/6j x CBA/Ca) mice aged 5–8 weeks were daily injected subcutaneously with a GnRH agonist (triptoreline, IPSEN, Belgium) or an antagonist (cetrorelix, Merck Serono, Belgium) at doses of 2, 20, 200 (4 animals per condition) and 500μg/kg (3 animals par condition), or with saline solution (10 animals) for 15 days. Four mice were also given a single im dose of 4mg/kg triptoreline (slow release) or saline solution. Mice were sacrificed after 15 days of treatment and the ovaries were removed for further immunohistological analysis.

In order to test the efficiency of GnRHa to protect ovarian reserve, GnRHa (500μg/kg) were administered for 21 days and Cy (200 mg/kg) or saline solution was injected ip on day 15 in three mice per group. During all experiments, mice were daily observed to evaluate the healthy status.

### Vaginal smears

Vaginal smears of treated mice were daily performed by gently flushing the vagina with 20μl of phosphate buffered saline (PBS). The oestrus cycle stage was defined using vaginal cytology analysis following Byers’s recommendations [30]. Briefly, pro-oestrus is characterized by a majority of nucleated cells and some cornified epithelial cells. At the oestrus stage, numerous cornified epithelial cells are present. The metoestrus stage, where the three cell types are present, is followed by the dioestrus stage, recognizable by the presence of mostly leukocytes and some nucleated cells. The cycle repeats each 4–5 days. The presence of oestrus cycle was verified at least 5 days before each injection.

### Histology and follicular count

The ovaries were fixed overnight in paraformaldehyde 4%, and embedded in paraffin. Microtome serial sections of 5μm were performed on the whole ovary. One slice out of 5 was stained and observed under light microscope for morphology and follicular staging following Gougeon’s classification [31]. Every 25 μm, primordial, primary, secondary, early antral and antral follicles with visible nucleoli into the oocyte were counted. Primordial and primary follicles were defined as the resting pool while secondary, early antral and antral follicles were considered as growing follicles. No correction factor was applied.

### TdT-mediated dUTP-biotin nick end labelling (TUNEL)

Apoptosis was assessed by TUNEL staining (In Situ Death Cell Detection Kit, Roche, Belgium). Sections of treated mice ovaries were deparaffinized, rehydrated and made permeable by proteinase K treatment (20μg/ml in Tris 10mM pH7.4, Qiagen, Netherlands). After washing, sections were labelled with TUNEL reagents according to manufacturer’s instructions (Roche) and counterstained with Hoechst (1μg/ml). Sections were observed using a Leica DM 2000 fluorescent microscope.

### Immunohistochemistry

Fixed ovarian sections were deparaffinized and rehydrated in order to evaluate the follicular proliferation (KI-67) and apoptosis (CASPASE-3) in treated mice ovaries. Antigen retrieval by heat was performed in citrate buffer pH6.0. For KI-67, the endogenous peroxidases were inhibited by hydrogen peroxide 1% (Merck Millipore, Belgium). Non-specific sites were blocked by normal goat serum and the slides were incubated with the primary antibody (KI-67 mouse α-human 1:400, BD Bioscience, Belgium or CASPASE-3 rabbit α-human 1:1000, Cell Signaling, Netherlands) overnight at 4°C. For KI-67, sections were incubated in secondary biotinylated goat anti-mouse antibody (1:300, Vector Laboratories, UK), and processed using an ABC kit according to manufacturer’s instructions (Vectastain Elite ABC systems, Vector Laboratories, UK). The reaction was revealed with diaminobenzidine (DAB Peroxidase Substrate Kit, Vector Laboratories, UK) followed by counterstaining with haematoxylin. For CASPASE-3, the slides were incubated with a biotinylated goat anti-rabbit secondary antibody (1:300, Vector Laboratories, UK), then with a fluorescein isothiocyanate (FITC) coupled avidin (1:200, Vector Laboratories, UK) followed by counterstaining with Hoechst. As a negative control, primary antibodies were replaced by species-adapted non-specific IgG. Sections were examined using a Leica DM 2000 fluorescent microscope.

### Follicle-stimulating hormone assay

Blood samples from GnRHa treated mice were collected by terminal cardiac puncture under anaesthesia [ketamine 75 mg/kg (Ceva, Belgium) and Rompun 10 mg/kg (Bayer, Belgium)]. The serum was separated by centrifugation and stored at -20°C until assayed with the immunofluorometric assay (IFMA) method at the Biomedicine Institute from University of Turku, Finland [32].

### Statistical analysis

All statistical analyses were performed with the SPSS program. The mean of each follicular stage counted were compared by a one-way ANOVA test, after a preliminary Levene’s test. Significance was confirmed when p < 0.05.

## Results

### Cyclophosphamide induced a dose-dependent and acute follicular loss

**Fig 1:**
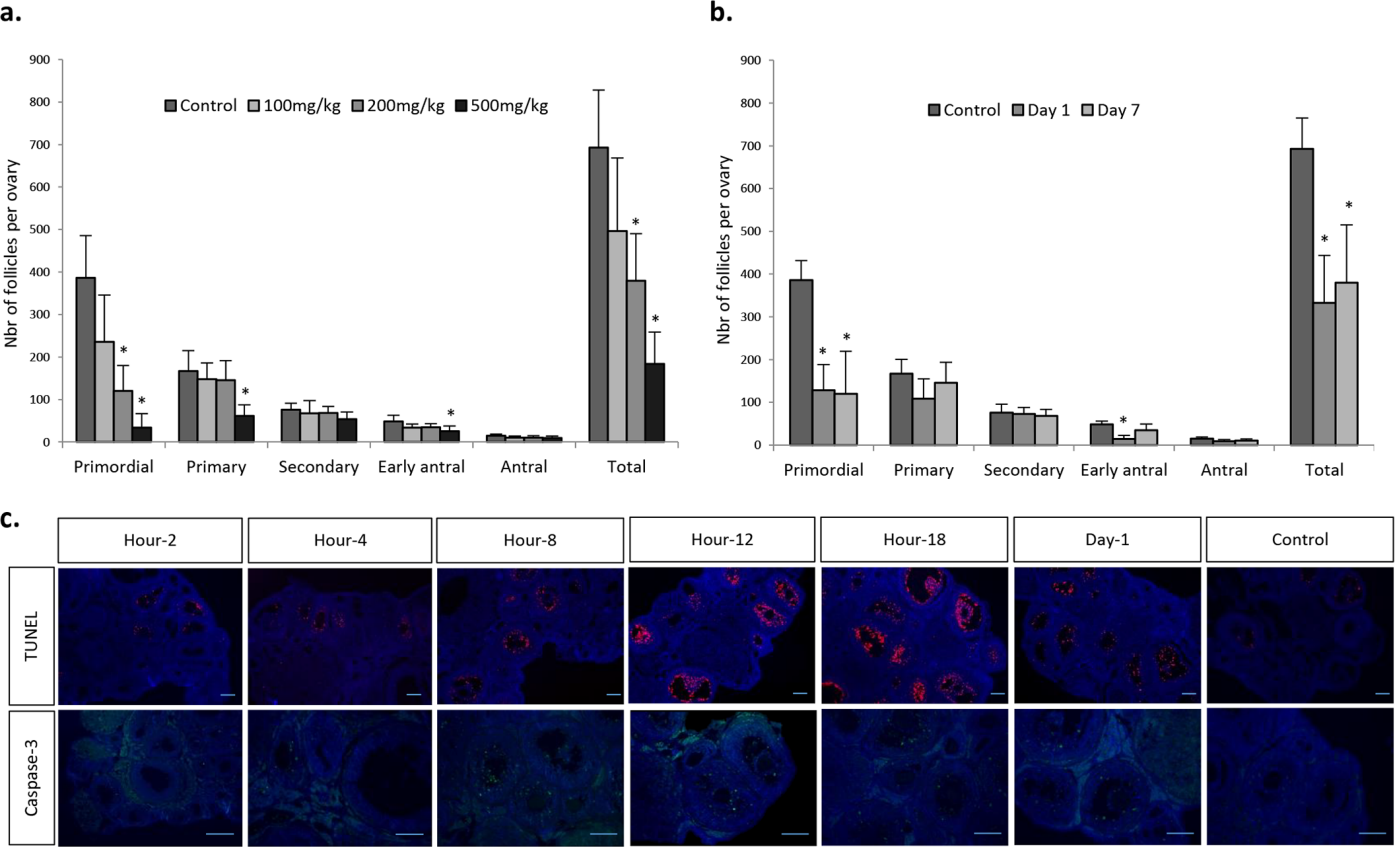
Dose- and time-related follicular depletion induced by chemotherapy. The follicular population was evaluated by counting all the follicles in every fifth section of each whole serial sectioned ovary from mice treated with (a) 100, 200, or 500 mg/kg of Cy and sacrificed after 7 days or (b) 200 mg/kg of Cy and sacrificed after 1 or 7 days. Results are expressed as mean ± SD; *p < 0.05 compared with controls. (c) Representative immunohistological sections showing apoptotic follicles (stained by TUNEL and CASPASE-3) in 200mg/kg Cy-treated mice sacrificed at different time points. TUNEL staining peaked at 12–18 hours, whereas CASPASE-3 peaked at 8–12 hours. Scale bar = 100 μm.

Mice were treated with different doses of Cy (100, 200, 500 mg/kg) or saline solution and sacrificed 7 days after injection. As expected, Cy induced a dose-dependent follicular depletion (Fig 1A). All follicular stages were affected, but the depletion was mainly observed in the primordial follicular pool. The total follicular loss reached 28.40% (p = 0.247), 45.19% (p < 0.01), and 73.45% (p < 0.001) for Cy doses of 100, 200, and 500 mg/kg, respectively, compared with the control (Table 1). Resting follicle population loss reached 51.91% (p = 0.01) with 200 mg/kg Cy, while only 18.59% of growing follicles were destroyed compared with the control. We observed an increase in the proportion of growing follicles compared with the resting population, as the proportion of growing follicles reached 23.21%, 31.40%, and 49.16% for chemotherapy doses of 100, 200, and 500mg/kg, respectively, compared with 20.68% for the control (Table 1).

**Table 1:** Total follicular depletion, and proportion of resting and growing follicles population per ovary according to the Cy doses in mice.

Cy dose	N	Total follicular loss (%)	Distribution of follicles	
			Number of resting follicles (%)	Number of growing follicles (%)
Control	6	–	553.2±130.5 (79.3)	139.8±27.8 (20.7)
100 mg/kg	6	28,40	383.8±141.7 (76.8)	112.3±35.4 (23.2)
200 mg/kg	6	45,19*	266.0±96.5 (68.6)	113.8±15.1 (31.4)
500 mg/kg	7	73,45*	95.7±43.6 (50.8)	88.3±32.6 (49.2)

Results are expressed as mean ± SD. N = number of ovaries analysed.

*p < 0.05 compared with controls.

**Fig 2:**
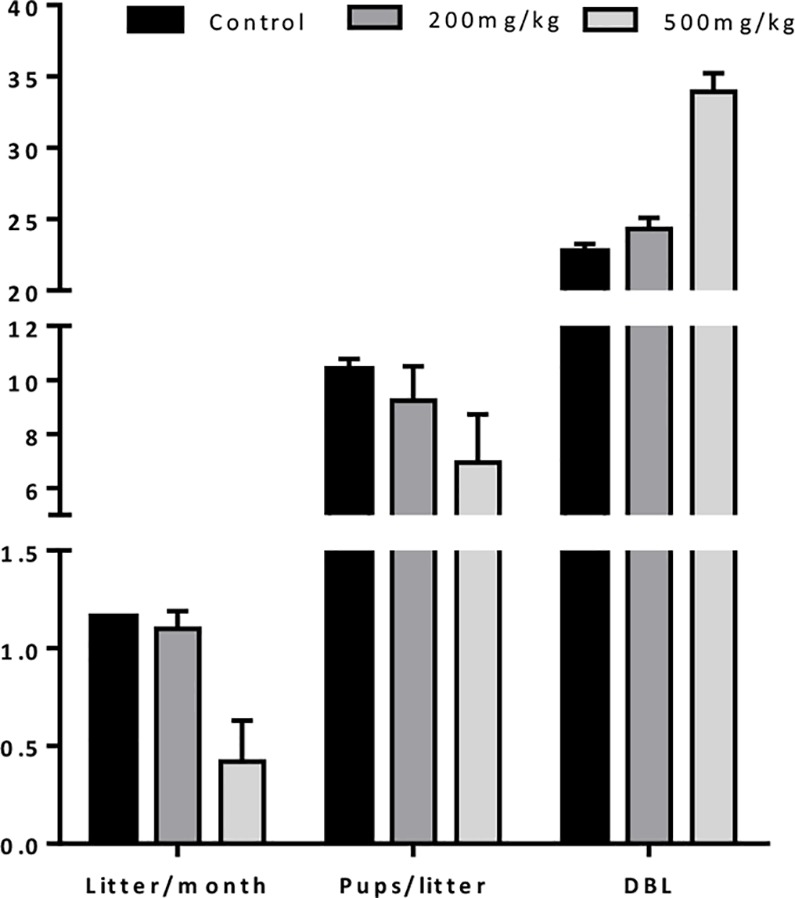
Fertility impact of chemotherapy. Mice were treated with Cy (200 or 500mg/kg) or saline and mated for 36 weeks. The number of litter per month, of pups per litter and the number of days between two consecutive litters (DBL) were calculated. Results are expressed as mean ± SD; N = 2 mice per condition.

The toxic effect of Cy on fertility was assessed by mating female mice treated with 200mg/kg or 500mg/kg of Cy for 36 weeks. The experiment was aborted at 19 weeks for the highest dose because of healthy issues (Fig 2). The experiment was not repeated due to ethical consideration. A dose of Cy of 200 mg/kg was chosen for the next experiment. This dose reduced the ovarian reserve of around 50% without significantly affecting fertility.

In order to evaluate the kinetics of chemotherapy-induced ovarian damage, mice were sacrificed at different times after Cy administration. Follicular loss was observed as early as 24 hours following Cy exposure (p = 0.001), affecting mainly the resting follicles (57.16%, p < 0.01) (Fig 1B). As follicles were early depleted, we further evaluate the time of apoptosis occurrence after Cy exposure (1, 2, 4, 8, 12, and 18 hours, 1, 2, 7, 14, and 21 days to cover the whole folliculogenesis process). TUNEL staining appeared in the granulosa cells after 4 hours and was maximal after 18 hours. The CASPASE-3 staining followed the same kinetics, with an earlier peak after 12 hours (Fig 1C). Staining for apoptosis returned to similar intensity as control at 7 days post-treatment. No apoptosis was observed in primordial and primary follicles, whatever the timing of observation.

### GnRHa partially blocked oestrus cycle but not folliculogenesis

**Fig 3:**
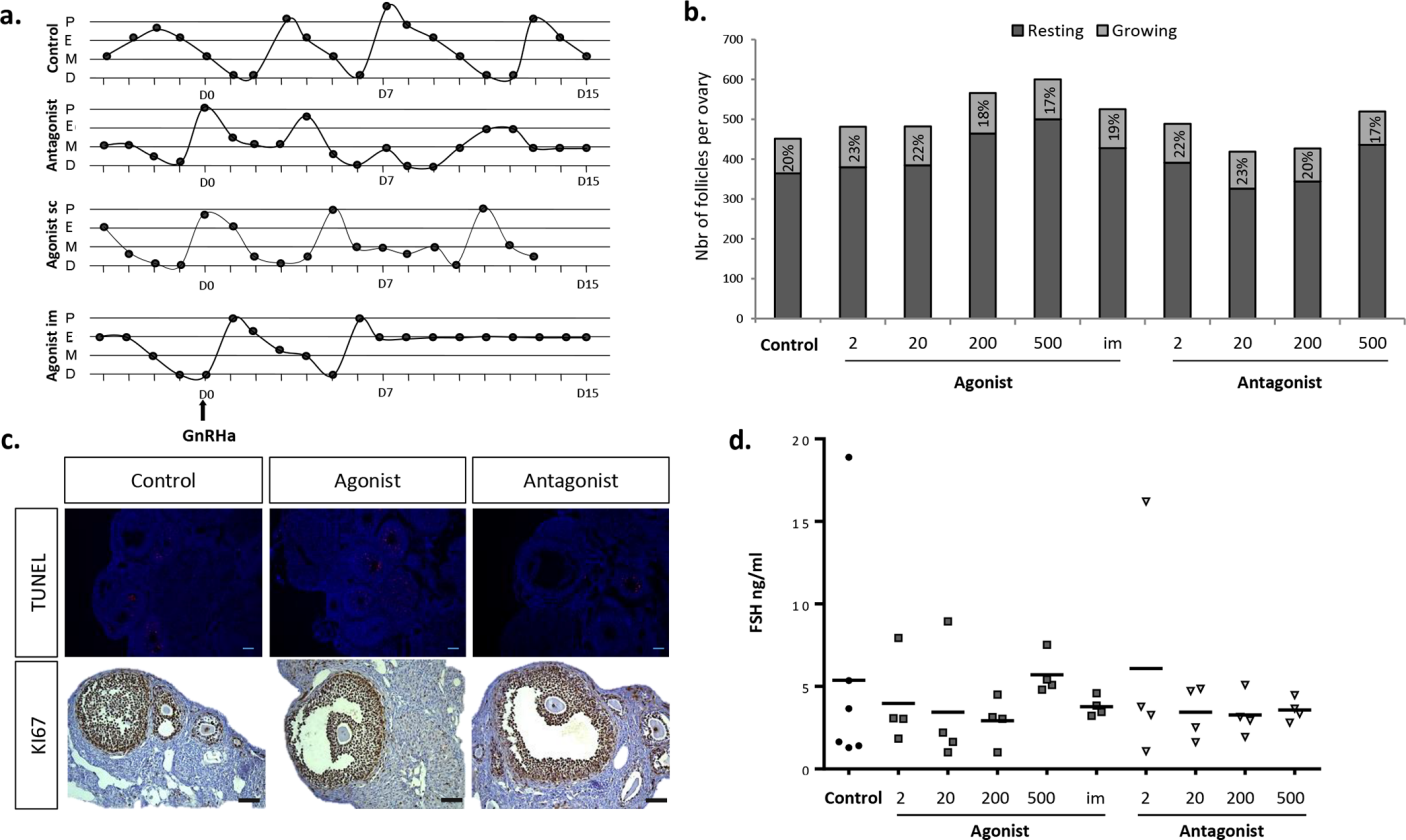
Inhibitory effect of GnRHa on follicular development. (a) The oestrus stages were determined by evaluating the vaginal cytology (pro-oestrus (P), oestrus (E), metoestrus (M) and dioestrus (D). The oestrus cycle is partially blocked according to the type of GnRHa, irrespective to the dose. Here oestrus cycle during treatment with antagonist 20μg/kg, agonist 2μg/kg and agonist im 4mg/kg are represented (b) The growing follicles ratio was evaluated by counting follicles in every fifth section of each whole serial sectioned ovary from different experimental conditions (2, 20, 200 or 500 μg/kg/day or im agonist injection of 4mg/kg). Results are expressed as mean ± SD. (c) Representative immunohistological sections showing growing follicles (stained with KI-67) without apoptosis staining in granulosa cells (TUNEL) after 15 days of GnRHa treatment. Scale bar = 100 μm. (d) Serum FSH levels (ng/ml) according to the doses and injection site of GnRHa. Each symbol represents one animal.

In order to evaluate the effect of GnRHa on ovarian function, we verified their inhibitory effect on FSH-dependent follicular development and on FSH secretion. Different doses of GnRH agonist or antagonist were tested, from a clinical-like dose of 2μg/kg/day to a higher dose previously used in rodent model (500μg/kg/day) [12, 33], and daily injected subcutaneously for 15 days (n = 4 per group). Four mice also received one intramuscular (im) injection of GnRH agonist (4mg/kg). Vaginal smears were daily performed to check the oestrus cycle phase. At least one complete oestrus cycle had to be observed before treatment. Representative profiles of oestrus cycles during treatment with GnRHa are illustrated in Fig 3A. Throughout the duration of treatment, 89% (8/9) of the control mice presented regular oestrus cycles (>3 cycles). In contrast, all GnRH agonist-treated mice (either sc or im; 17/17) displayed less than three cycles, compared with only 58.30% (7/12) in the GnRH antagonist-treated mice (Fig 3A).

We further examined the distribution of follicular stages in the ovary from GnRHa-treated mice. The follicular distribution was not affected by treatments whatever the dose, injection site, and type of GnRHa. The percentage of growing follicles ranged from 16.50% to 23.25% in the treated groups and was not significantly different from the control (20.40% ± 4.16%) (Fig 3B). Furthermore, KI-67 and TUNEL staining revealed that growing follicles still contained proliferating cells and no difference was observed in the apoptosis pattern (Fig 3C). In accordance with this observation, serum FSH levels were not reduced in GnRHa-treated mice (Fig 3D).

### GnRHa failed to protect the ovary against Cy-induced follicular depletion

**Fig 4:**
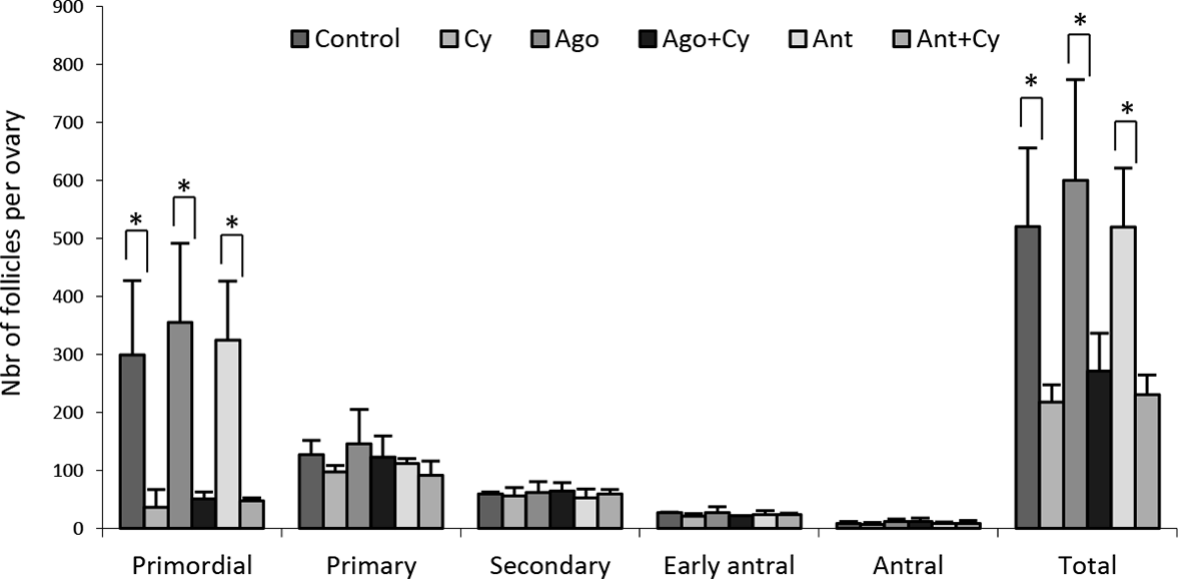
Evaluation of the protective effect of GnRHa on Cy-induced follicular depletion. The follicular population was evaluated by counting all follicles in every fifth section of whole serial sectioned ovaries of mice injected daily with 500μg/kg of GnRH agonist or antagonist subcutaneously for 21 days and treated with 200mg/kg of Cy on day 15. The different conditions are control, Cy alone (Cy), GnRH agonist alone (Ago), or with Cy (Ago + Cy) and GnRH antagonist alone, (Ant) or with Cy (Ant + Cy). Results are expressed as mean ± SD, *p < 0.05 compared with controls. Statistical difference was observed between Cy-treated groups and control but not with GnRHa treatments.

Three mice per group were treated with GnRH agonist or antagonist (500μg/kg/day) and Cy (200mg/kg). The number of follicles, the apoptosis, and proliferation in the ovaries, as well as the oestrus cycle were assessed. As expected, we observed a significant depletion of total follicles of 56.24% ± 1.81% after Cy administration (Table 2). Concomitant treatment with GnRHa did not prevent reduction of the ovarian reserve (Fig 4). A decrease in the proportion of resting follicles in favour of the growing pool was observed after chemotherapy, and this was not altered by treatment with GnRHa (Table 2). TUNEL and KI-67 staining did not reveal any differences between the treatments (data not shown).

**Table 2:** Total follicular depletion, and proportion of resting and growing follicles population per ovary according to the treatment in mice.

Treatment	N	Total follicular loss (%)	Distribution of follicles	
			Number of resting follicles (%)	Number of growing follicles (%)
Control	3	–	426.0±135.0 (81.05)	94.7±5.1 (18.95)
Cy	3	58,26*	133.7±41.1 (60.83)	83.7±20.6 (39.17)
Ago	3	–	500.7±142.6 (83.50)	99.3±34.3 (16.50)
Ago + Cy	3	54,78*	173.7±48.0 (63.64)	97.7±20.4 (36.36)
Ant	3	–	435.7±104.2 (83.30)	84.0±13.0 (16.70)
Ant + Cy	4	55,68*	139.0±23.43 (60.28)	91.3±12.9 (39.72)

Cy single ip injection of 200 mg/kg; GnRH agonist (Ago) daily subcutaneous (sc) injection of 500 μg/kg; GnRH antagonist (Ant) daily sc injection of 500 μg/kg. Results are expressed as mean ± SD. N = number of ovaries analysed.

*p < 0.05 compared with controls.

## Discussion

In order to elucidate the controversial question of ovarian protection by GnRHa during chemotherapy and validate the model, the physiological effect of GnRH analogues at different dosages was assessed to evaluate their ability to inhibit the hypothalamic–pituitary–ovarian axis in this commonly used animal species.

Here we showed a toxic effect of Cy on both the resting and growing follicular population in mice at different times, irrespective to the administration of GnRH analogue. In accordance with previous work [34], 200mg/kg of Cy destroyed around 50% of the ovarian follicle pool, without affecting fertility. At the dose of 500mg/kg, Cy dramatically affected the ovarian reserve, the fertility and health of the mice, increasing the mortality rate. This dose has been previously described as lethal in 50% of mice [34]. While it is assumed that chemotherapy affects the ovarian reserve, the mechanisms of direct or indirect damage remains unclear. We confirmed that Cy induces acute apoptosis of granulosa cells in growing follicles within 24 hours of administration. Primordial follicles were destroyed by chemotherapy as early as day 1 but no apoptosis was observed in this pool, suggesting different actions of Cy on resting and growing populations. Previous study using an in vitro model showed that the toxicity of Cy on primordial follicles was independent of CASPASE activation [35]. Recently, Morgan *et al*. [36] observed primordial follicles loss induced by cisplatin or doxorubicin but did not bring evidence of apoptosis in primordial follicles after 24 hours exposure. Other studies have shown that doxorubicin caused apoptosis in stroma and granulosa cells within 12 hours, while apoptotic events were not detected in primordial follicles until 48 hours post-injection [37]. These results suggested that acute primordial follicle loss in response to chemotherapy drug exposure was not due to apoptosis. However, primordial follicles of p63-deficient mice, as well as PUMA- and/or noxa-deficient mice, are resistant to γ-radiation, cisplatin, and Cy [38, 39]. The direct effect of Cy on the primordial pool was also suggested in humans. In a xenograft model of ovarian pieces from aborted foetuses containing only resting follicles, Oktem and Oktay [5] showed that Cy reduced the primordial follicle density and increased TUNEL staining, which peaked at 12 hours.

The dose-dependent increase in the proportion of growing follicles observed after Cy exposure can be explained by the activation of resting follicles or by a higher sensitivity of those compared with the growing pool. Our observations support the hypothesis of the early direct effect of chemotherapy on primordial follicles, independent of the ‘burn out’ effect. However, we cannot exclude later ovarian damage due to apoptosis of granulosa cells in growing follicles. The decrease in the derived paracrine growth factors secreted by granulosa cells as AMH may induce a massive recruitment of primordial follicles into the growing pool after chemotherapy exposure [6]. Destroying the growing follicular pool also removes the negative feedback on the pituitary gland, leading to an increase in gonadotropins levels. In humans, Cy exposure resulted in a rapid decline in AMH level associated with an increase in FSH level [40, 41]. However, apoptosis was reported in granulosa cells of growing follicles at 7 days post-injection in mice, although AMH tissue expression or serum level were not reduced [33]. A recent study showed that Cy itself may activate the PI3K pathway, which has been shown to trigger follicle activation [8, 42].

While the mechanisms of GnRHa ovarian protection are still unclear, the main hypothesis is that the inhibition of FSH secretion makes follicles at the resting stage less vulnerable to chemotherapy. Other possible actions include a decrease in utero-ovarian perfusion, the up-regulation of anti-apoptotic molecules or, more recently, by directly inducing an increase of AMH in granulosa cells [43]. In mice, several studies have shown a reduction of chemotherapy-induced follicular depletion when associated with these drugs [12, 13, 33, 43]. Others did not show any evidence for ovarian protection or even toxic action [20–22]. Although pituitary inhibition is the main hypothesis for ovarian protection during chemotherapy, very few studies have investigated the GnRHa effect on folliculogenesis in a rodent model. Here, we showed that GnRH agonist and antagonist were not efficient in blocking gonadotropin secretion and follicular development, irrespective of the doses tested. GnRHa disrupt the oestrus cycle with a greater efficacy for the agonist than for the antagonist. This difference can be explained by different mechanisms of action of agonists and antagonists to down-regulate pituitary GnRH receptors [29]. A delay before inhibition was observed in the agonist group due to the flare-up effect. Despite GnRHa treatment, the proportion of healthy growing follicles was similar in all groups, as well as the FSH level. While the suppression effect of GnRHa on gonadotropin secretion was clearly demonstrated in humans [44, 45], this is the first study analyzing the effect of GnRHa on folliculogenesis, oestrus cycle and pituitary suppression in the mice model. The limitation of this study is the single mice strain used for experiments but it is unlike that the absence of inhibition of the follicular growth is specific to this strain. It was previously reported that GnRH antagonist blocked ovulation in treated ovaries in rat [46] but induced only oestrus cycle perturbation in C57BL mice [47]. Singh & Krishna [48] did not observe any marked change in vaginal cytology after 8 days of 1–25μg/day of GnRH agonist treatment in Parkes mice. When associated with Cy, GnRH antagonist limited the increase of FSH level in rats [49] while agonist implant did not in mature C57BL mice [21]. Studies did not report any change in FSH level in rats treated with GnRHa [15, 49], while others showed a decrease in GnRH pituitary receptors and oestradiol levels [28, 29]. A recent study showed a decrease in FSHb and LHb mRNA expression in the pituite of ICR mice treated with GnRH agonist but the impact on circulating hormone levels and follicular growth was not described [43]. Finally, FSH level is less affected by GnRHa than LH level [45], which may explain the inhibitory effect of the drug on oestrus cycles but not on follicular growth.

In this study, we provide accurate data on folliculogenesis dynamics during GnRHa and Cy treatment, essential for the interpretation of experiments on fertility preservation using mice model. We showed that GnRHa disturbed the oestrus cycle, but did not block folliculogenesis. GnRHa administration did not inhibit the pituitary–gonadal axis in mice as effectively as it does in humans. Hence, the mouse model may not be appropriate for the investigation of the mechanisms of GnRHa protection by suppressing the hypothalamic–pituitary–ovarian axis. Finally, Cy induces severe acute damages in primordial follicular pool, suggesting that the inhibitory effect of GnRHa on pituitary gonadotropin secretion may not be sufficient to protect the ovarian reserve against chemotherapy damage. Direct protective effect of GnRHa on the ovary cannot be excluded but the mechanisms of action of GnRHa through the ovarian receptor should be further investigated using in vitro model.
